# Digital home monitoring in patients with juvenile idiopathic arthritis to increase intervals of hospital visits

**DOI:** 10.1093/rheumatology/keag444

**Published:** 2026-09-15

**Authors:** Anne Jasmijn Sellies, Joeri W van Straalen, Gerrie C J de Joode-Smink, Martijn J H Doeleman, Marc H A Jansen, Erika Van Nieuwenhove, Annet van Royen-Kerkhof, Bas J Vastert, Nico M Wulffraat, Sytze de Roock, Joost F Swart

**Affiliations:** Department of Paediatric Immunology and Rheumatology, Wilhelmina Children’s Hospital, University Medical Centre Utrecht, Utrecht, The Netherlands; Faculty of Medicine, Utrecht University, Utrecht, The Netherlands; Department of Paediatric Immunology and Rheumatology, Wilhelmina Children’s Hospital, University Medical Centre Utrecht, Utrecht, The Netherlands; Faculty of Medicine, Utrecht University, Utrecht, The Netherlands; Department of Paediatric Immunology and Rheumatology, Wilhelmina Children’s Hospital, University Medical Centre Utrecht, Utrecht, The Netherlands; Faculty of Medicine, Utrecht University, Utrecht, The Netherlands; Department of Paediatric Immunology and Rheumatology, Wilhelmina Children’s Hospital, University Medical Centre Utrecht, Utrecht, The Netherlands; Faculty of Medicine, Utrecht University, Utrecht, The Netherlands; Department of Paediatric Immunology and Rheumatology, Wilhelmina Children’s Hospital, University Medical Centre Utrecht, Utrecht, The Netherlands; Faculty of Medicine, Utrecht University, Utrecht, The Netherlands; Department of Paediatric Immunology and Rheumatology, Wilhelmina Children’s Hospital, University Medical Centre Utrecht, Utrecht, The Netherlands; Faculty of Medicine, Utrecht University, Utrecht, The Netherlands; Department of Paediatric Immunology and Rheumatology, Wilhelmina Children’s Hospital, University Medical Centre Utrecht, Utrecht, The Netherlands; Faculty of Medicine, Utrecht University, Utrecht, The Netherlands; Department of Paediatric Immunology and Rheumatology, Wilhelmina Children’s Hospital, University Medical Centre Utrecht, Utrecht, The Netherlands; Faculty of Medicine, Utrecht University, Utrecht, The Netherlands; Department of Paediatric Immunology and Rheumatology, Wilhelmina Children’s Hospital, University Medical Centre Utrecht, Utrecht, The Netherlands; Faculty of Medicine, Utrecht University, Utrecht, The Netherlands; Department of Paediatric Immunology and Rheumatology, Wilhelmina Children’s Hospital, University Medical Centre Utrecht, Utrecht, The Netherlands; Faculty of Medicine, Utrecht University, Utrecht, The Netherlands; Department of Paediatric Immunology and Rheumatology, Wilhelmina Children’s Hospital, University Medical Centre Utrecht, Utrecht, The Netherlands; Faculty of Medicine, Utrecht University, Utrecht, The Netherlands

**Keywords:** disease activity, patient-reported outcomes, delivery of health care, organization of health care, outcome measures, quality of health care, telemedicine, interventional studies, juvenile idiopathic arthritis, paediatric/juvenile/child/adolescent

## Abstract

**Objectives:**

Juvenile Idiopathic Arthritis (JIA) patients attend routine hospital check-ups, even when disease activity is low. These visits are time-consuming and costly. Replacing visits with home monitoring may reduce costs but should not affect safety and (perceived) quality of care. This non-inferiority study tested whether patients with inactive JIA could safely prolong visit-intervals by home monitoring disease activity using EuroQol EQ-5D-Y-5L and Juvenile Arthritis Multidimensional Assessment Report questionnaires.

**Methods:**

One regular 3-monthly visit was replaced by home monitoring. Healthcare professionals evaluated responses to determine whether patients could safely prolong their visit-interval or needed a doctor’s consultation. Flare percentages over the total study period and 6 months after baseline were compared with a historical flare percentage and with matched JIA patients using relative risks (RR) and a non-inferiority margin of 15%. Secondary outcomes were number of reminders, number of patients not completing home monitoring and patient satisfaction.

**Results:**

Eighty-four patients aged between 6 and 20 years participated in the study. Eighteen patients had a visit before or after evaluation of the questionnaires. Sixty-three had a prolonged interval, of whom seven experienced a flare. The study-wide flare percentage was 18.5%, the historical flare percentage was 18.4% (including non-inferiority margin). The RR for study-wide flares was 0.50 (95%CI: 0.29–0.86), showing non-inferiority. The study was underpowered for comparing flares after prolonged visit-intervals (RR: 0.77, 0.30–1.93). Overall, 92% showed interest in home monitoring more often.

**Conclusion:**

No clear evidence was found that increased visit-intervals with home monitoring poses a risk for missing disease flares. Reminders were necessary and patient satisfaction was high.

**Trial registration:**

Clinicaltrials.gov.uk; NCT05603286.

*Rheumatology* key messages

- This non-inferiority study shows no clear safety risks in replacing a hospital visit with home monitoring using the EuroQol EQ-5D-Y-5L and Juvenile Arthritis Multidimensional Assessment Report (JAMAR) questionnaires.
- Home monitoring may safely reduce the number of hospital visits, saving time and costs.
- Patient satisfaction with home monitoring is high, but home monitoring requires reminders to complete questionnaires and flexibility for scheduling early in-person visits if needed.

## Introduction

Juvenile idiopathic arthritis (JIA) is the most common chronic rheumatic disease in childhood and may cause severe disability and a reduced quality of life [1–3]. Treatment includes immunosuppressive drugs for reducing arthritis, which are commonly prescribed in a step-up approach [4–6]. Due to therapeutic advances in the last three decades, such as the availability of methotrexate and biologicals, inactive disease and remission have become realistic goals for most children with JIA [2, 7]. Following a treat-to-target strategy, the main aim of JIA treatment is to achieve and sustain the target of inactive disease or at least minimal levels of disease activity using regular clinical assessments of disease [8, 9]. In addition, recent approaches emphasize the timely initiation of biological Disease-Modifying Anti-Rheumatic Drugs (bDMARDs) to achieve rapid disease control [9, 10]. A commonly used disease activity measure in this treat-to-target approach to treating JIA is the clinical Juvenile Arthritis Disease Activity (cJADAS) score [11, 12], which takes into account the number of joints with active arthritis, a Physician Global Assessment of disease activity and a patient or parent assessment of well-being.

JIA patients commonly visit a paediatric rheumatologist at regular intervals to monitor disease activity, medication-related side effects, growth and milestone development. Visit schedules may vary between countries and clinical settings. Occasionally, patients with inactive disease have less frequent visits. Control visit intervals also had to be increased during the peak of the COVID-19 pandemic. The availability of paediatric rheumatologists is limited, leading to challenges in timely access to care and highlighting the need for more efficient care strategies [13, 14]. Systematically increasing the visit interval of JIA patients with inactive disease might be safely facilitated by remote self-monitoring of disease activity using patient-reported outcomes. In previous studies, four selected JIA-specific parent/child-reported outcome measures for disease activity (parent/patient assessment of pain, disease activity level, joints with active arthritis and morning stiffness) showed good criterion validity and excellent reliability and could be considered for remote patient assessment [15]. In addition, moderate to high disease activity in JIA patients could be identified by remote self-monitoring with the EuroQol five-dimensional youth questionnaire with five levels (EQ-5D-Y-5L) [16].

Although measures that can be useful to home monitor JIA patients have been identified, a study testing the safety and feasibility of remote monitoring has not been conducted yet. For this reason, the aim of this study was to assess non-inferiority of home monitoring with these previously identified patient-reported outcomes compared with regular 3-monthly outpatient clinic visit.

## Methods

In this single-centre, non-inferiority trial, the study cohort of JIA patients was compared with a historical flare percentage and a cohort of matched historical control JIA patients. Participants in the study cohort and data of historical controls were included from the Paediatric Immunology and Rheumatology department of the Wilhelmina Children’s Hospital, a tertiary care centre in Utrecht, the Netherlands. Participants were included between March and December 2022.

Inclusion criteria were a diagnosis of JIA for at least one year (all JIA subtypes were eligible), inactive disease defined by an active joint count (AJC) score of 0, and an age between 6 and 20 years. There were no restrictions regarding medication use for inclusion. Exclusion criteria were insufficient proficiency in the Dutch language and/or inability or unwillingness to use e-mail.

### Ethics approval and consent to participate

This study has been approved by the Medical Research Ethics Committee of the UMC Utrecht, the Netherlands (local IRB protocol number 21–767; National CCMO nr. NL78722.041.21 and ClinicalTrials.gov ID NCT05603286). All participants (and/or their parents) had to provide written informed consent to enter this study. The study is conducted according to the principles of the Declaration of Helsinki and in accordance with the WMO and ICH Good Clinical Practice guidelines.

### Study protocol

After inclusion, participants were scheduled to skip one regular 3-monthly control visit and instead complete an online Juvenile Arthritis Multidimensional Assessment Report (JAMAR) and EuroQol EQ-5D-Y-5L questionnaire [17, 18]. This could be completed together with a parent or guardian if needed.

Questionnaires were sent to the participants or a parent/guardian 11 weeks after the baseline visit via e-mail using Castor EDC (Electronic Data Capture) [19]. Completed questionnaires were evaluated by an experienced nurse of the paediatric rheumatology team, in consultation with the treating paediatric rheumatologist. In case there was suspicion of disease worsening based on the home monitoring results, the participant was called for additional questions and if needed scheduled for an outpatient visit as soon as possible. In case no suspicion of disease worsening arose, the participant was followed-up at the hospital 6 months (range 5–7 months) after the baseline visit. Patients who wished to visit the hospital anytime sooner could always contact their treating paediatric rheumatologist to do so.

### Objectives

The primary objective of this study was to investigate if replacing one outpatient visit with home monitoring is non-inferior to regular 3-monthly visits with regard to disease flares in the first 6 months after baseline. Secondary objectives were (i) to investigate the number of early scheduled hospital visits, (ii) to investigate the number of patients that needed reminders or did not complete home monitoring questionnaires and (iii) to investigate patient satisfaction with home monitoring as an alternative for visiting the hospital.

### Intervention and outcome definition

The intervention (exposure) of interest was defined as increasing the hospital control visits interval from 3 to 6 months, with substitutive home monitoring. This home monitoring consisted of the EuroQol EQ-5D-Y-5L and the JAMAR questionnaire, which could be completed within a range of 11–14 weeks after baseline.

The outcome of a disease flare was defined as an AJC of 1 or higher. The paediatric rheumatologist recorded a cJADAS (including AJC) and possible AEs at the intended follow-up (5–7 months after inclusion), which was after skipping one 3-monthly control visit, or at a prior clinical visit if this was deemed necessary by the patient or hospital team.

Furthermore, participants were requested to complete a short five-item questionnaire to indicate their satisfaction with home monitoring, including one dichotomous (yes/no) item, three ordinal items with five-point response scales and one open-text field for comments and suggestions.

### Safety assessment

#### Flare percentage historical cohort

To evaluate the safety of this protocol in daily clinical practice, the percentage of flares in the study cohort was compared with a historical flare percentage. For this, all patients were selected, regardless of JIA subtype and medication. As calculated from historical data from 2461 unique visits in 542 unique patients between January 2015 and December 2019, a disease flare was observed in 16.0% of the visits after a JIA patient was evaluated to have clinically inactive disease at the previous visit, between 30 days and 210 days before. The median visit interval in the historical data was 105 days (IQR: 84–144 days).

#### Flares compared with matched historical control group

To assess a relative risk (RR), a comparison was made to a matched group based on age and medication, expected to be more comparable to the study cohort.

#### Non-inferiority margin (Δ)

The non-inferiority margin (Δ) was set to 0.15, allowing for up to 18.4% (15% more) flares in the study population compared with the historical cohort; this margin was approved by the Institutional Review Board Utrecht and was considered clinically acceptable by paediatric rheumatologists in the study centre, considering that disease can result from multiple factors, may not directly cause joint damage and can sometimes go unnoticed by patients.

### Statistical methods

#### Matching

1:1 matching was performed on the study participants using controls from a historical cohort. Matching was based on age and medication. No maximum allowable difference (caliper) was applied. Matching was conducted using nearest neighbour matching without replacement for each patient, based on propensity score distances.

For the matching process, the same inclusion criteria as for the study population were used; inactive disease (defined as an AJC of 0), and 6–20 years of age. Additionally, only visits before 2020 were considered for matching. The reason for this time span is 2-fold: avoid overlap with the study period and avoid the influence of the COVID-19 pandemic, which may have resulted in missed outpatient visits. Furthermore, it was required that AJC data for the historical controls were available at baseline, 3 and 6 months, ensuring no visits were missed.

#### Sample size calculation

As calculated beforehand, a total sample size of 170 patients (85 study participants) would give the study a statistical power of 80% to establish non-inferiority for a margin of 15% more disease flares in the study cohort compared with the matched historical cohort.

#### Statistical analysis

Baseline characteristics were compared between the study population and the matched historical control group using *χ*^2^ tests or Fisher’s exact tests when expected cell counts were small. Bonferroni correction was applied to adjust for multiple comparisons. Differences in age and follow-up time between the two groups were assessed using the Mann–Whitney *U* test.

To evaluate non-inferiority, the percentage of disease flares in the study cohort was compared with the percentage of disease flares calculated from the historical patients including the non-inferiority margin being 18.4% [=16.0% historical flares × 15% (Δ)] and compared with the historical matched control group using RRs with 95% confidence intervals (CIs). Flare percentages below 18.4% were considered non-inferior, as well as a 95% CI of the RR entirely below the cut-off of 1.15.

The remaining secondary outcomes were analysed using descriptive statistics. Statistical analyses were carried out using R version 4.2.2 [20]. Matching balance diagnostics were performed using the ‘cobalt’ package (bal.tab function) to evaluate the success of matching [21]. The sample size was calculated using the ‘SampleSize4ClinicalTrials’ package [22].

## Results

Twelve eligible patients refused participation, of whom nine stated that they preferred not to replace an in-person visit with home monitoring. In total, 87 participants were included in the study. Three participants did not meet the inclusion criteria and were excluded, leading to an enrolled study cohort of 84 at baseline (Supplementary Fig. S1).

### Baseline characteristics

The median age in the study population was 14.2 years (IQR: 11.9–16.7) and the majority of participants were female (63%) (Table 1). Most included patients had the oligoarticular subtype of JIA (50%) and did not receive any systemic medication, or used NSAID only (36%). The patients in the study cohort were matched to historical patients at baseline (Table 1). Matching diagnoses showed balanced measures for age and medication, with standardized mean differences below 0.1.

**Table 1:** Baseline characteristics of patients included for home monitoring and patients in the matched historical control group.

Baseline characteristics	Study cohort (home monitoring) (*n* = 84)	Matched historical control group (*n* = 84)	*P*-value
Age in years, median (IQR)	14.2 (11.9–16.7)	14.6 (12.1–17.0)	0.78
Sex (female), *n* (%)	53 (63%)	55 (66%)	0.87
JIA subtype, *n* (%)			0.22
Oligo arthritis	42 (50%)	29 (35%)	*0.43*
RF − polyarthritis	13 (16%)	17 (20%)	*>0.9*
Psoriatic arthritis	10 (12%)	6 (7%)	*>0.9*
Enthesitis-related arthritis	9 (11%)	11 (13%)	*>0.9*
Systemic arthritis	7 (8%)	15 (18%)	*0.77*
RF + polyarthritis	2 (2%)	3 (4%)	*>0.9* ^a^
Undifferentiated arthritis	1 (1%)	3 (4%)	*>0.9* ^a^
Medication use, *n* (%)			0.81
No medication/NSAID	30 (36%)	22 (26%)	*>0.9*
Mono therapy			
csDMARD	12 (14%)	12 (14%)	*>0.9*
Anti-TNF therapy	11 (13%)	12 (14%)	*>0.9*
Other targeted therapy	8 (10%)	8 (10%)	*>0.9*
Combination therapy			
csDMARD & Anti-TNF therapy	21 (25%)	28 (33%)	*>0.9*
csDMARD & Other targeted therapy	2 (2%)	2 (2%)	*>0.9* ^a^
Follow-up time in days, median (IQR)	182 (154–196)	183 (98–196)	0.46

The study cohort represents patients included for home monitoring. The control group consisted of patients selected from databases with historical data. Patients in the control group were matched to patients in the study group. Values in italics represent statistical tests of categories within the parent variable.

bDMARD: biological disease-modifying antirheumatic drug; csDMARD: conventional synthetic disease-modifying antirheumatic drug; IQR: interquartile range; JIA: juvenile idiopathic arthritis; *n*: number; NSAID: non-steroidal anti-inflammatory drugs; RF: rheumatoid factor.

Bonferroni correction was applied to adjust for multiple comparisons.

a Fisher’s Exact test.

Three participants were lost to follow-up for various reasons, including changing hospitals and other logistical factors (Supplementary Fig. S1). Additionally, one patient was included twice in the study due to initially not following the protocol, but later complying. Both participations for this patient were included in the analyses.

### Flares in study population

At the end of the study, 15 patients in the total study population of 81 patients (18.5%) had experienced a disease flare anywhere during the study period (Table 2). In the total study population, 18 had an early scheduled visit before the anticipated prolonged 6-months visit. For eight of these 18 patients with early scheduled visits the physician observed a disease flare (44.4%). The remaining 63 patients in the study cohort had a prolonged visit interval and visited the hospital after 6-months. At this visit, seven patients had a disease flare (11.1%).

**Table 2:** Incidence of flares in study population.

Analysis	Number of patients	Number of flares	Flare percentage
All flares within total study population	81	15	18.5%
Flares within patients with early scheduled visits	*18*	*8*	*44.4%*
Flares within patients with a prolonged visit interval	*63*	*7*	*11.1%*
Flares after a prolonged visit interval within total study population	81	7	8.6%

Percentages of all flares in total study population, divided in flares within patients with early scheduled visits (before 6 months) (*n* = 18), and flares in the population with an actual prolonged visit interval (*n* = 63) and flares after a prolonged visit interval within the total study population (*n* = 81). The historical flare percentage including non-inferiority margin (Δ of 15%) was 18.4%. Values in italics represent values for the subgroups within ‘All flares within the total study population’.

In the historical data that were used for comparison, the mean percentage of flares was 18.4% (including non-inferiority margin), with a median interval between visits of 105 days.

### Relative risk for flares

The RR of flares in the study participants compared with matched historical control patients was 0.50 (95% CI: 0.29–0.86) for the study-wide flares (31 flares in 84 patients), and 0.77 (95% CI: 0.30–1.93) for the patients with a prolonged visit interval (9 flares in 62 patients) (Fig. 1, Supplementary Table S1).

**Figure 1:**
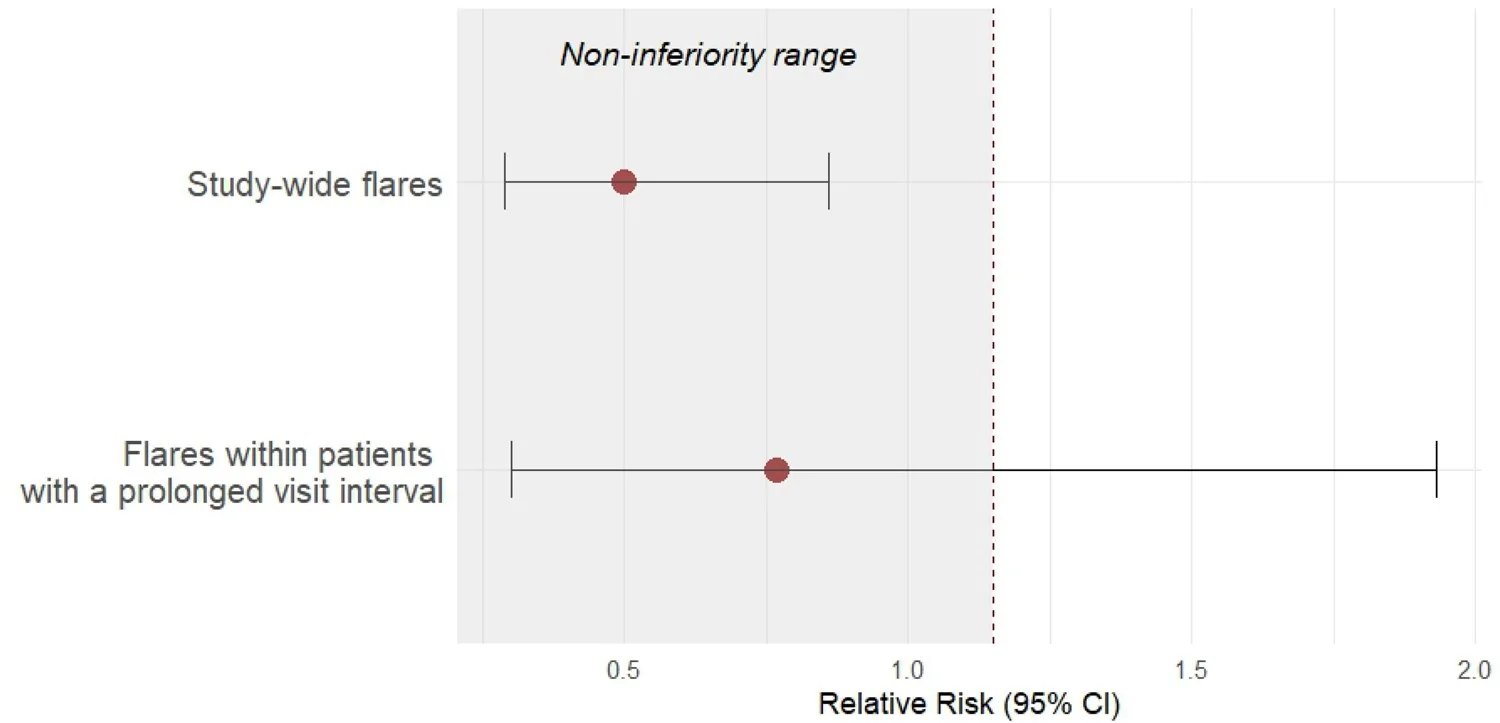
Relative risks with 95% CIs of flares in study population and matched control group for all flares within the total study population and flares within patients with a prolonged visit interval. The non-inferiority margin (Δ) is set at 15%, with values below the red lined cut-off of 1.15 (1 + Δ) indicating non-inferiority, displayed as the grey area. CIs with an upper bound crossing the cut-off are considered inconclusive

For the study-wide flares comparison, the RR was non-inferior (95% CI upper limit did not exceed the cut-off of 1.15). Non-inferiority was not demonstrated for patients with a prolonged visit interval; the 95% CI exceeded the non-inferiority cut-off of 1.15.

### Early return visits

Of the 18 participants with an early scheduled visit, 13 had requested to reschedule the hospital visit themselves, of which 7 patients did so before home monitoring with flares observed in 42.9% and 6 patients after home monitoring with 50% presenting a flare (Fig. 2). The study team scheduled another five hospital visits based on home monitoring results, with flares observed in 40%.

**Figure 2:**
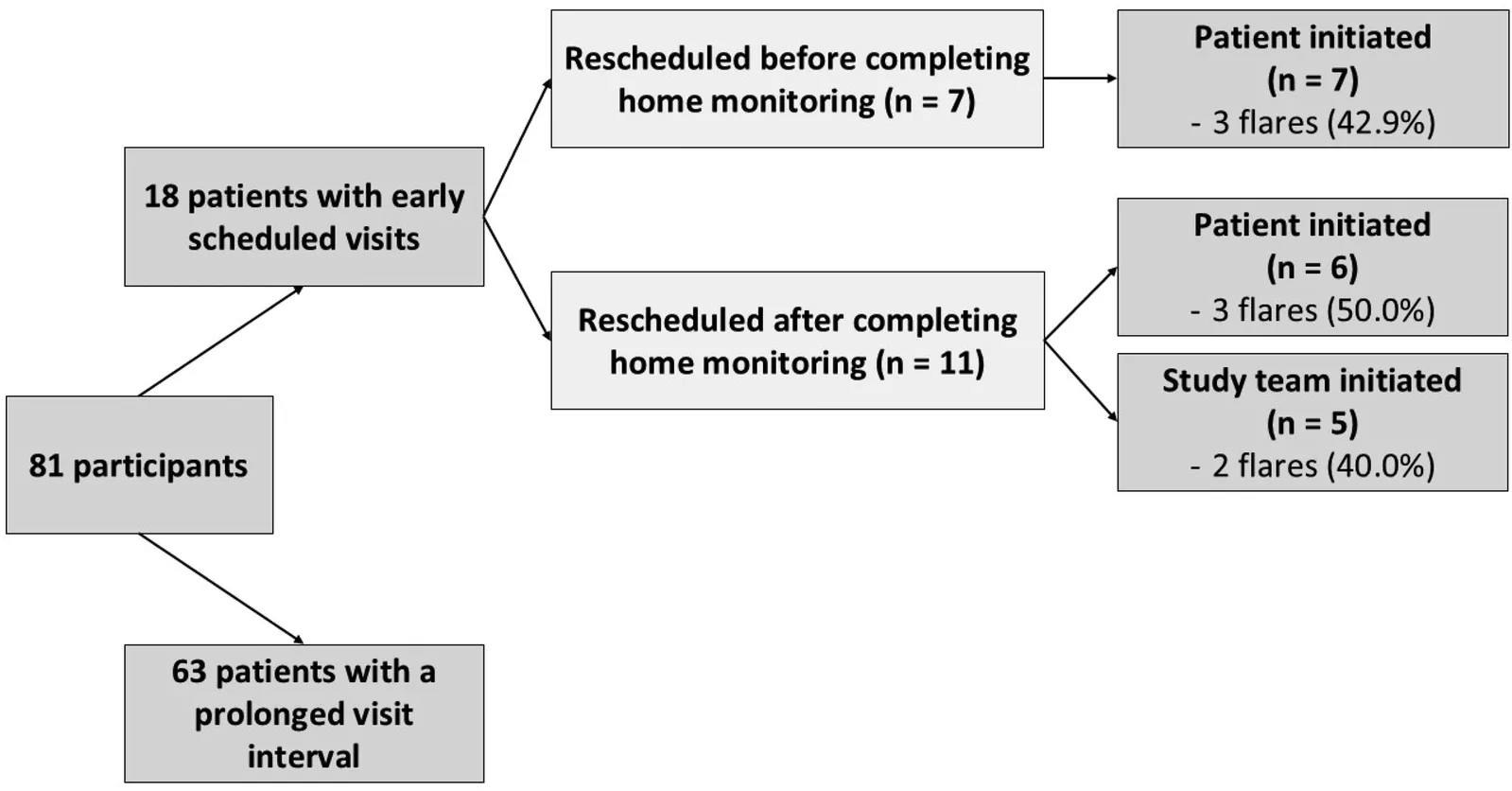
Number of early scheduled visits, number of flares at early scheduled visits and number of visits after an actual prolonged visit interval

### Home monitoring

Home monitoring questionnaires were sent to 77 participants; seven of the 84 participants already had an early return visit before the home monitoring questionnaires were sent out. Twenty-three participants (30%) did not need a reminder for filling out the questionnaires (Fig. 3). Twenty-five participants (32%) had to be reminded once (email), 27 (35%) participants had to be reminded twice (email and telephone) and two participants failed to complete (i.e. did not submit home monitoring results, even after two reminders).

**Figure 3:**
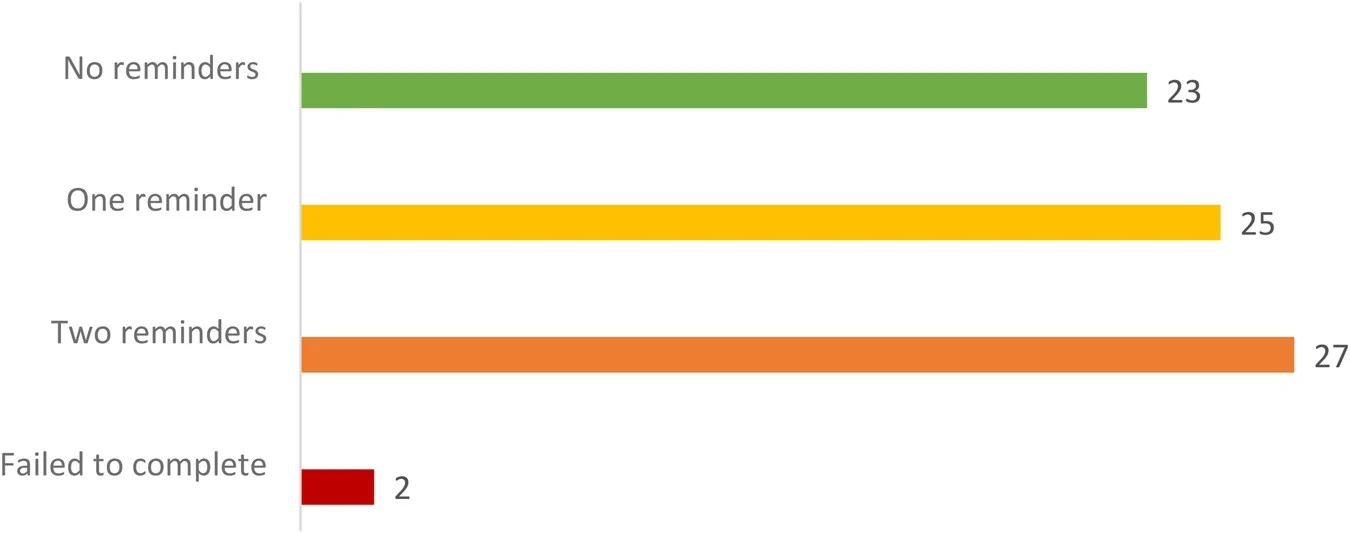
Reminders needed for home monitoring and failure to home monitor. 77 participants received the home monitoring questionnaires; 7 of 84 participants had an early scheduled visit before the home monitoring questionnaires were sent out. The first reminder was over email, and the second over telephone

### Patient satisfaction and adverse events

All participants were asked to complete a patient satisfaction questionnaire after participation. Sixty participants completed the patient satisfaction questionnaire, of whom 59 (98.3%) indicated that they felt safe or very safe during the study, and 55 (91.7%) indicated that they would like to skip a hospital control visit more often using home monitoring (Supplementary Fig. S2).

During follow-up, 17 patients reported adverse events, with COVID-19 infections being the most frequently reported (four times).

### Flares

The majority of flares had a low AJC (1–2), with one isolated case reaching an AJC of 5. Within the patients experiencing a flare during the study period (*n* = 15), eight patients received an intra-articular joint injection with corticosteroids. Additionally, six patients underwent a change in medication or dosage; two patients have received both interventions (joint injection and change in medication/dosage).

Of the patients who were observed with a flare after home monitoring or at the 6-month visit (*n* = 12), six (50%) completed home monitoring questionnaires after receiving a reminder over telephone and five (42%) with a reminder only over email, or no reminder. One additional patient did not complete home monitoring and experienced a flare (AJC = 5), detected at the 6-month follow-up.

One participant had the 6-month visit at 8.2 months, exceeding the range of 5–7 months. This participant had an AJC of 1 at the follow-up visit.

## Discussion

This non-inferiority study examined if the time interval between hospital control visits can be safely increased for JIA patients with inactive disease by home monitoring disease activity using online EuroQol EQ-5D-Y-5L and JAMAR questionnaires, comparing a study group with a historical flare percentage and a matched control group.

Previous studies have explored the use of digital health tools in JIA, mainly focusing on the monitoring of symptoms and quality of life, self-management and the monitoring of physical activity, for example through the use of apps or wearable devices [23, 24]. Parameters that can be assessed remotely and show a good correlation with disease activity may be useful for home monitoring. Recent research has shown that the EQ-5D-Y-5L can help identify patients with higher disease activity who may require treatment adjustments [16]. In addition, specific symptoms such as morning stiffness, as assessed in the JAMAR questionnaire, have been suggested as potential markers for remote disease monitoring [25]. While these studies demonstrate the feasibility and potential benefits of digital health interventions, most focus on supporting or complementing routine care and do not specifically address the replacement of routine in-person visits. In adult rheumatology, a systematic review has shown that telemedicine can achieve outcomes comparable to in-person care while reducing the number of outpatient visits [26]. Comparable evidence in paediatric rheumatology cohort studies remains limited, highlighting the relevance of the present study.

JIA requires long-lasting complex care, which poses a considerable economic burden on patients, parents/caregivers, healthcare providers and insurance companies [27–29]. Remote monitoring of children with chronic disease has the potential of reducing these costs as a result of a decrease in hospital visits [30]. This will also remove part of the burden on paediatric rheumatology units, especially in areas with few available paediatric rheumatologists. As a consequence, physicians can allocate more time and effort to complex and urgent cases. Most importantly, patients who can safely home monitor themselves will miss less days of school and their parents or guardians will miss less days of work, benefiting families and society in general.

The expected risk of any articular or extra-articular damage while skipping one hospital visit in this study is negligible due to several measures. First, only patients with inactive disease at baseline were considered eligible for a prolonged visit interval. This group of patients has a small chance of developing damage in the 6 months of the study. Second, an increase in disease activity should be reflected in the home monitoring results and thus be observed by the study team in a timely manner. Previous research demonstrated that self-assessment with the EuroQol EQ-5D-Y-5L can distinguish between inactive arthritis and moderate to high disease activity with high discriminatory power [16]. Third, patients can always call their treating physician for advice or schedule a control hospital visit if needed.

One of the main limitations of this study was the small sample size of the study population. Our initial sample size calculation indicated that 85 participants were needed to achieve 80% power; we were able to include 84 patients, with three lost to follow-up. While we had planned to use cJADAS to assess disease activity, a higher proportion of missing cJADAS data led us to switch to AJC as the outcome measure (33% missing cJADAS at baseline in the study population due to 33% missing Patient Global Assessment). Therefore, the probability of observing a disease flare, originally based on historical data using cJADAS and used for the sample size calculation, no longer fully aligned with the final analysis. As a result, the study was underpowered, resulting in inconclusive findings. This limitation affects the robustness of the conclusions. In addition, the limited sample size did not allow for meaningful subgroup analyses, and larger studies are needed to explore potential differences between patient subgroups such as JIA subtypes and age-groups.

Although the study population was limited, no indications were observed that the protocol with prolonged hospital intervals was unsafe. Implementation of this protocol in clinical practice is not expected to result in unexpected events. Nevertheless, close monitoring of the number of flares in patients that home monitor themselves is advisable.

Another limitation was the difference in time since the last in-person visit between the intervention group and the historical data, which may have affected the comparison of flare percentages. The 16% flares calculated from the historical data corresponded to a median of 105 days (IQR: 84–144 days) since the last visit, whereas in the intervention group this period was extended to a median of 182 days (IQR: 154–196 days) due to the nature of the intervention, likely increasing the flare risk. However, this was addressed in the matched control analysis, where both the intervention group and matched control group had a comparable follow-up period [median time control group 183 days (IQR: 98–196 days)].

Despite balance measures appearing adequate, the matched control group may be subject to some selection bias, as the data were primarily derived from patients who had a recorded AJC available in their electronic health records. Patients with more severe disease may be more likely to attend follow-up appointments and thus to be included in the control group, potentially skewing flare percentages. These factors suggest that the control group may not fully reflect the whole JIA population.

The findings do not suggest a risk, as flares in the study group were not significantly higher than in the control group and the severity was low (low AJC). However, careful consideration is needed before implementing this protocol. Apart from the intention to assess its safety, the study’s goal was to improve efficiency and reduce workload. However, in reality, 27 patients (35%) had to be reminded over email and telephone before they filled out the questionnaires, which was time consuming for the study nurse. When implementing this protocol, this needs to be taken into account. Time spent on reminders and reviewing questionnaire results was not formally recorded, limiting comparison of time investment between home monitoring and in-person hospital visits. Additionally, in case 3-monthly blood checks are required, this needs to be factored in as well. Patient satisfaction based on the questionnaire was generally high. However, this may be biased, as twelve patients refused to participate and not all participants completed the satisfaction survey. Additionally, patient preferences, such as a possible reluctance to prolong hospital visit intervals, should be considered when implementing such measures.

In conclusion, the findings indicate no clear evidence that replacing one hospital control visit with home monitoring poses a significant risk for JIA patients with inactive disease. There was a high questionnaire response, although reminders and early scheduled visits were necessary. Patient satisfaction with home monitoring was high.

## Supplementary Material

keag444_Supplementary_Data

## Data Availability

The data supporting this study are available upon reasonable request. Due to the clinical and sensitive nature of the data, only a limited and de-identified dataset can be shared.
