# Supplementary material for: Digital home monitoring in patients with juvenile idiopathic arthritis to increase intervals of hospital visits
Source: Rheumatology (Oxford). 2026 Sep 15;65(10):keag444. doi: 10.1093/rheumatology/keag444 (PMC13649185; doi:10.1093/rheumatology/keag444)
Supplement: Supplementary file 1 [file keag444_supplementary_data.docx]

# **Resu****lts – Supplementary material**

**Supplementary Table S1.** Relative risks (RR) with 95% confidence intervals (95% CI) of flares in study population and matched control group.

| **Risk comparison** | **Flares in study cohort** | **Flares in matched historical controls** | **RR (95% CI)** | **Non-inferiority^a^** |
| --- | --- | --- | --- | --- |
| *All flares* | 15 / 81 | 31 / 84 | 0.50 (0.29 – 0.86) | Non-inferior |
| *Flares within patients with a prolonged visit interval* | 7 / 63 | 9 / 62 | 0.77 (0.30 – 1.93) | Inconclusive |

^a^ The non-inferiority margin (Δ) is set at 15%, with values below the cut-off of 1.15 (1+Δ) indicating non-inferiority. Confidence intervals with an upper bound crossing the cut-off are considered inconclusive.

**Supplementary Figure S1.** Overview of participant inclusion and follow-up.

**Supplementary Figure S2.** Results of patient satisfaction questionnaires, n=60. Questions in the questionnaire were: 1) How easy was it for you to complete the questionnaires from home? 2) How safe did you feel during the study? 3) How satisfied are you about home monitoring? 4) Would you like to skip a hospital visit using home monitoring more often?
